# Oculocardiac Reflex

**Published:** 1916-04

**Authors:** 


**Oculocardiac Reflex.** Levine, Arch. of Internal Med., May 15, 1915.

1. Ocular pressure affords a simple and safe method of obtaining reflex vagus inhibition of the heart.

2. Inhibition of the heart by the oculocardiac reflex is much more profound and more frequently obtained than by pressure over the vagus nerve.

3. The oculocardiac reflex is generally absent in tabes dorsalis, present in pneumonia, syphilis (non-tabetic), and chronic valvular disease.

4. The reflex was absent in one case of diabetes mellitus and also in one case of auricular fibrillation before digitalis treatment. It was present after digitalis was given.

5. Right ocular pressure has a slightly greater effect on the rate of the heart than left. It may stop the heart for a long period of time, relatively speaking; the P-waves are sometimes diminished in size and may become iso-electric. Occasionally the auriculoventricular interval is lengthened.

6. Left ocular pressure has a much greater effect on the conduction mechanism of the heart than right. It may lengthen auriculoventricular conduction, cause partial heart-block, and result, possibly, in automatic ventricular rhythm. On two occasions inverted P-waves were seen. The height of the R-waves is sometimes increased, at other times diminished. Ectopic ventricular beats were twice observed. The P-waves are often diminished in size, but occasionally are increased. Escaped ventricular beats were seen both during right and left ocular pressure.

7. Pain, flushing of the face, and apnea during ocular pressure, are much less pronounced in tabetics than in non-tabetics.

8. The effects on the rate and on the rhythm of the heart produced by ocular pressures and not constant, differing in different individuals and in the same individual from time to time. The duration and degree of pressure play an important part in the degree of inhibition.

